# A comprehensive comparison of the *in vitro* hemocompatibility of extracorporeal centrifugal blood pumps

**DOI:** 10.3389/fphys.2023.1136545

**Published:** 2023-05-09

**Authors:** Ping Li, Xu Mei, Wanning Ge, Tingting Wu, Min Zhong, Nana Huan, Qiubo Jiang, Po-Lin Hsu, Ulrich Steinseifer, Nianguo Dong, Liudi Zhang

**Affiliations:** ^1^ Department of Cardiovascular Surgery, Union Hospital, Tongji Medical College, Huazhong University of Science and Technology, Wuhan, China; ^2^ Artificial Organ Technology Lab, Biomanufacturing Centre, School of Mechanical and Electrical Engineering, Soochow University, Suzhou, China; ^3^ Department of Cardiovascular Engineering, Institute of Applied Medical Engineering, Helmholtz Institute, Medical Faculty, RWTH Aachen University, Aachen, Germany

**Keywords:** centrifugal pump, ECMO, hemolysis, von Willebrand factor, blood damage

## Abstract

**Purpose:** Blood damage has been associated with patients under temporary continuous-flow mechanical circulatory support. To evaluate the side effects caused by transit blood pumping, *in vitro* hemocompatibility testing for blood damage in pumps is considered a necessary reference before clinical trials.

**Methods:** The hemocompatibility of five extracorporeal centrifugal blood pumps was investigated comprehensively, including four commercial pumps (the Abbott CentriMag, the Terumo Capiox, the Medos DP3, and the Medtronic BPX-80) and a pump in development (the magAssist MoyoAssist^®^). *In vitro*, hemolysis was tested with heparinized porcine blood at nominal operating conditions (5 L/min, 160 mmHg) and extreme operating conditions (1 L/min, 290 mmHg) using a circulation flow loop. Hematology analyses concerning the blood cell counts and the degradation of high-molecular-weight von Willebrand factor (VWF) during 6-h circulation were also evaluated.

**Results:** Comparing the *in vitro* hemocompatibility of blood pumps at different operations, the blood damage was significantly more severe at extreme operating conditions than that at nominal operating conditions. The performance of the five blood pumps was arranged in different orders at these two operating conditions. The results also demonstrated superior hemocompatibility of CentriMag and MoyoAssist^®^ at two operating conditions, with overall low blood damage at hemolysis level, blood cell counts, and degradation of high-molecular-weight VWF. It suggested that magnetic bearings have an advantage in hemocompatibility compared to the mechanical bearing of blood pumps.

**Conclusion:** Involving multiple operating conditions of blood pumps in *in vitro* hemocompatibility evaluation will be helpful for clinical application. In addition, the magnetically levitated centrifugal blood pump MoyoAssist^®^ shows great potential in the future as it demonstrated good *in vitro* hemocompatibility.

## 1 Introduction

Centrifugal blood pumps have been widely used, as ventricular assist devices (VADs) or as part of the extracorporeal membrane oxygenation (ECMO) circuit, in extracorporeal life support to drive blood in the circuit (Bottrell et al., 2014; Wu et al., 2021a). As a temporary continuous-flow mechanical circulatory support device, a centrifugal blood pump is considered clinically favorable (Nishida et al., 1993; Bennett et al., 2004). Despite the effectiveness of centrifugal blood pumps in prolonging the lives of heart failure patients, it has been reported that patients being bridged could develop a series of blood damage-related clinical hemocompatibility complications (Eckman and John, 2012; Kirklin et al., 2017). The complications could be the consequence of the side effect of pre-sheared blood in the blood pumps (Borchardt et al., 2012; Sun et al., 2020). When heart failure patients are treated with VADs, high-level non-physiological shear stress created by the pumps can damage blood components, including blood cells and protein in the plasma (Fraser et al., 2012; Chen et al., 2019).

To evaluate these side effects, hemocompatibility testing for overall blood damage caused by a centrifugal blood pump is considered a necessary reference before clinical trials. *In vitro* and *in vivo* tests are two commonly used ways of evaluating the hemocompatibility of blood pumps. However, scientific head-to-head comparisons cannot be made using a few samples because animal blood and hemodynamic differences can cause pumps to operate at different conditions. Thus, while performing a head-to-head hemocompatibility comparison test, an *in vitro* test is also recommended in preclinical studies. Using the *in vitro* hemolysis test, the red blood cell trauma caused by a developing blood pump can be quantitatively analyzed and evaluated, referring to key indicators in terms of the normalized hemolysis index (NIH) and modified hemolysis index (MIH) (Sobieski et al., 2012). Many studies demonstrated that compared to mechanical bearings, blood pumps using non-contacting bearings with advanced technologies like hydrodynamics suspension and/or magnetic levitation could alleviate damage to blood components (Ranganath et al., 2020; Wu et al., 2021b). In practice, blood damage-associated adverse events, e.g., hemolysis and in-pump thrombosis, were reduced (Kosaka et al., 2009; Bourque et al., 2016). The incidence of clinical complication gastrointestinal (GI) bleeding remains high (Bansal et al., 2019), which has been proven as a consequence of the loss of high-molecular-weight von Willebrand factor (VWF) after continuous-flow mechanical circulatory support in clinics (Stockschlaeder et al., 2014; Bartoli et al., 2015). Therefore, it is necessary to involve VWF damage in the hemocompatibility test of centrifugal blood pumps.

Although extracorporeal centrifugal blood pumps were initially designed for a specific nominal operating condition, they could be manipulated into a broader operation range in a clinic due to the various settings, the resistance of different pipelines, or under the worst-case clinical scenario (Schöps et al., 2021). Moreover, neonatal or pediatric patients receiving ECMO and patients undergoing extracorporeal CO_2_ removal represented a lower blood flow rate than conventional ECMO (Schöps et al., 2021). It has been verified that blood damage in blood pumps varies widely under different operating conditions (Gross-Hardt et al., 2019; Kuck et al., 2021). In particular, adverse events such as hemolysis, clotting, and GI bleeding complications were frequently reported at low blood flow rate operations (Gross-Hardt et al., 2019; Schöps et al., 2021). Hence, the overall blood damage in centrifugal blood pumps at a more comprehensive operating condition range should also be investigated to provide a more thorough evaluation, especially the extreme work condition (low flow rate and high-pressure head) (Chan et al., 2015; Radley et al., 2019).

In this study, comprehensive perspectives on blood damage from five centrifugal blood pumps were investigated, including four commercial pumps and a pump in development. The comparison of three mainstream commercial extracorporeal blood pumps and a new fully maglev extracorporeal blood pump with the clinical standard CentriMag was conducted. The influence of different operating conditions on hemocompatibility was also evaluated. Here, we present our study on the *in vitro* hemolysis level of five blood pumps at nominal operating conditions (5 L/min, 160 mmHg) and extreme operating conditions (1 L/min, 290 mmHg) using a circulation flow loop. The 160-mmHg pressure head at nominal operating conditions was due to the resistance of the peripheral tubes used in clinical practice. The extreme operating condition was chosen to reflect the worst-case clinical scenario. Hematology analysis concerning change in the number of white blood cells and platelets over time was conducted. The degradation of high-molecular-weight VWF multimers during 6-h circulation was also evaluated.

## 2 Materials and methods

### 2.1 Device description

The following extracorporeal centrifugal blood pumps were included in this study: the CentriMag magnetically levitated centrifugal pump (Abbott, Thoratec, Pleasanton, CA, United States), the Capiox centrifugal pump (Terumo Corp., Tokyo, Japan), the Deltastream DP3 diagonal rotary pump (Medos Medizintechnik AG, Stolberg, Germany), the Bio-pump BPX-80 (Medtronic, Inc., Minneapolis, MN, United States), and the MoyoAssist^®^ device (magAssist., Inc., Suzhou, China). The Deltastream DP3 is a rotary blood pump with combined characteristics of an axial and a radial design. All the blood pumps are commercially used devices, except magAssist MoyoAssist^®^. MoyoAssist^®^ is a magnetically levitated extra-VAD that could provide bi-ventricular support for patients with acute heart failure (Li et al., 2022). Different from CentriMag and MoyoAssist^®^, Capiox, DP3, and BPX-80 are mechanical bearing centrifugal pumps. The maximum flow rate of CentriMag, BPX-80, and MoyoAssist^®^ is 10 L/min, while that of Capiox and DP3 is 8 L/min. The maximum operating pressures of CentriMag, Capiox, DP3, BPX-80, and MoyoAssist^®^ are 600 mmHg, 800 mmHg, 600 mmHg, 1,100 mmHg, and above 600 mmHg, respectively.

### 2.2 Flow loop design and protocol

**FIGURE 1:**
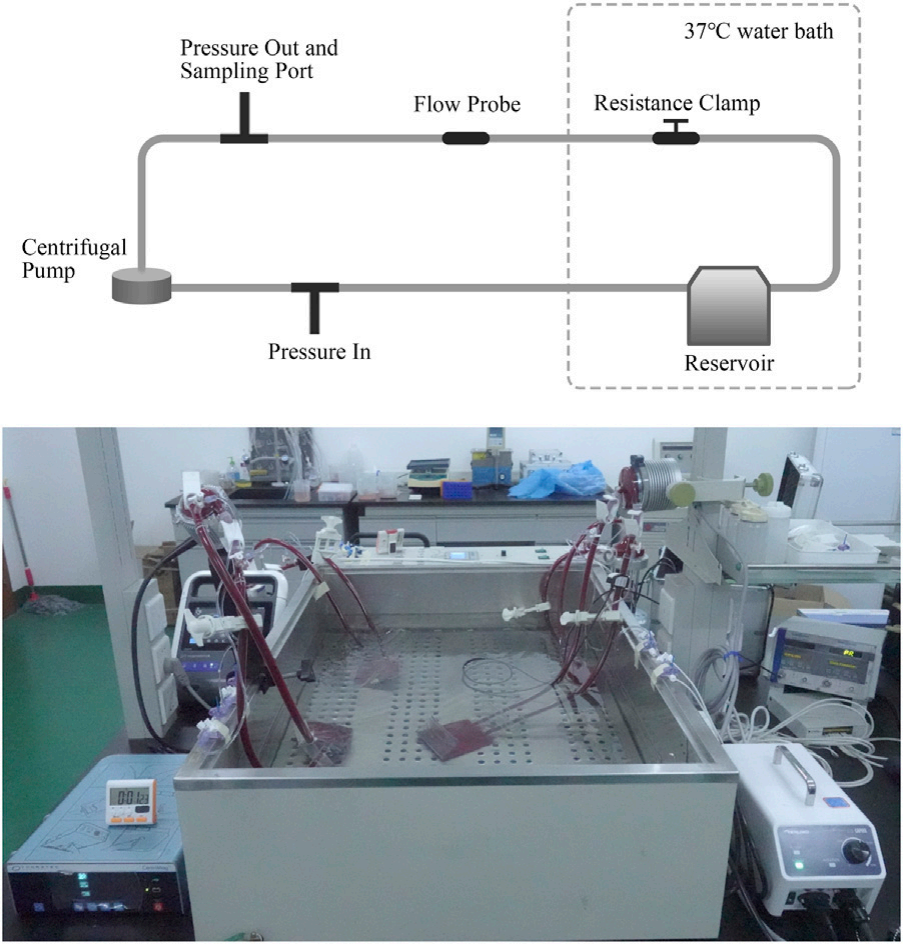
Schematic diagram and picture of the blood circulation loop. The loop setup allows for control of temperature, flow rate, and pressure head.

Domestic porcine blood with 0.4% heparin sodium anticoagulant was purchased from Suzhou Frankenman Medical Devices Co., Ltd. The activated coagulation time (ACT) of blood was kept greater than 300 s (ISO, 2016). The *in vitro* blood circulation loop was built referring to the American Society for Testing and Materials (ASTM) standard F1841-97 (Figure 1) (ASTM, 2019). For each set of tests, four loops were operated simultaneously, and the blood was taken from one pig. Due to the limited blood volume per porcine model, the loop was designed smaller than the ASTM standard, and the option of centrifuging blood for controlling the hematocrit was not performed. The hematocrit (Hct) of blood in the loop was controlled at 21% ± 4%, and the total blood volume was 310 ± 10 mL. The total hemoglobin at time zero was 8.3 ± 1.0 g/dL. The blood pumps were tested under stable hemodynamic conditions for 6 h on nominal (5 ± 0.25 L/min, 160 ± 5 mmHg) and extreme (1 ± 0.05 L/min, 290 ± 8 mmHg) operations, respectively. The flow rate was measured using an ultrasonic flow sensor (T402, Transonic Systems Inc., Ithaca, NY, United States). The differential pressure across the pump was measured using pressure sensors (DPT-01, Nora, Shenzhen, China). All the test loops and a separate static control blood bag were immersed in the thermostatic water bath to maintain a blood temperature of 37°C ± 1°C. During the experiment, blood samples were collected through the sample port at 0, 30, 60, 120, 180, 240, 300, and 360 min. The blood samples at time zero were obtained from blood that circulated for 5 min to ensure complete mixing. The first 2 mL of the blood sample was discarded, followed by a second volume of 3 mL. Blood samples from the static control blood bag were collected in the same way at 0, 120, 240, and 360 min.

### 2.3 Hemolysis measurement

Blood samples were centrifuged twice at ×1,500 g for 10 min, and the supernatants were collected to measure plasma-free hemoglobin. Samples were diluted 10 times with 0.01% Na_2_CO_3_ solution, and then, the triplicate was added to a 96-well labeled plate (Jet Biofil, Guangzhou, China). The absorbance was measured using the SpectraMax Paradigm Multi-Mode Microplate Reader (Molecular Devices, California, United States) at three wavelengths of 380 nm, 415 nm, and 450 nm. The absorbance of each sample at three wavelengths of 415 nm, 380 nm, and 450 nm was denoted as A_415_, A_380_, and A_450_, and the absorbance difference of each sample was calculated according to

$$Y=2A_{415}-A_{380}-A_{450}$$ (1)

where Y represents the absorbance difference of each sample. Then, the value Y was substituted back into the standard formula Y=0.0018Hb+0.0013 to calculate the plasma-free hemoglobin concentration. The NIH and MIH were calculated as follows:

$$NIH\;\left(g/100L\right)=∆pfHb\times V\times \frac{100-Hct}{100}\times \frac{1}{Q\times T\times 1000}$$ (2)

$$MIH=∆pfHb\times V\times \frac{100-Hct}{100}\times \frac{1}{Q\times T\times Hb}$$ (3)

Here,

ΔpfHb: increase of the plasma-free hemoglobin concentration (g/L)

Hb: total blood hemoglobin concentration at time zero (g/dL)V: blood volume in the loop (mL)

Q: flow rate (L/min)Hct: hematocrit (%)

T: sampling time (min)

### 2.4 Hematology analysis

The porcine blood samples from both the circulation loop and static control blood bag were collected for hematology analysis at time points 0, 120, 240, and 360 min. The total numbers of white blood cells and platelets in the samples were determined using the clinical automatic hematology system (ADVIA 2120i, Siemens Healthcare Diagnostics Inc., Erlangen, Germany).

### 2.5 Immunoblotting of VWF

Blood samples were centrifuged at 15,000 g for 5 min to collect the supernatant and remove debris. The VWF multimers in plasma samples were separated by gel-electrophoresis for 3.5 h and transferred onto a 0.45-µm polyvinylidene difluoride membrane (Immobilon-P; Millipore Corporation, Bedford, MA, United States) overnight. Then, primary antibody (Polyclonal Rabbit anti-Human von Willebrand factor, Cell Signaling Technology, Boston, United States) and secondary antibody (Polyclonal Rabbit anti-Rabbit lgG HRP-linked antibody, Massachusetts, United States) tests were performed to detect the VWF multimers’ molecular weight distribution on the film. The whole process included five main steps: SDS agarose gel preparation, electrophoresis, Western blot, immunolocalization, and visualization. After the visualization step, ImageJ software was used to process the VWF bands’ image obtained from the Kodak film (Rayco Medical Products Company Limited for Carestream Health, Xiamen, China) development. The high-molecular-weight VWF multimers section was selected to acquire the gray value corresponding to each sample. This value did not represent the actual molecular weight of VWF. The loss percentage of high molecular weight VWF multimers was expressed as

$$Degradation\;of\;high\;molecular\;weight\;VWF\;\left(\%\right)=\frac{H_{control}-H_{sample}}{H_{control}}\times 100$$ (4)

where H_sample_ is the gray value of high molecular weight bands corresponding to each blood sample and H_control_ is the gray value of high molecular weight bands corresponding to each blood sample at time zero.

### 2.6 Statistical analysis

Three valid measurements were obtained from repeated tests of the MoyoAssist^®^ and CentriMag centrifugal blood pumps. Due to the limitations of the experimental conditions, two accurate repeated measurements were obtained for BPX-80 on the nominal operation and Capiox on the extreme operation. Since DP3 and BPX-80 were only used for a single test on extreme operation, no statistical analysis was performed. The averages and standard deviations were calculated using data analysis software (Origin version 9, OriginLab, Northampton, MA, United States). Due to multiple measurements taken from each blood pump, statistical analysis was carried out using one-way ANOVA. A value of *p* < 0.05 (**) was considered to be statistically significant.

## 3 Results

### 3.1 Hemolysis

**FIGURE 2:**
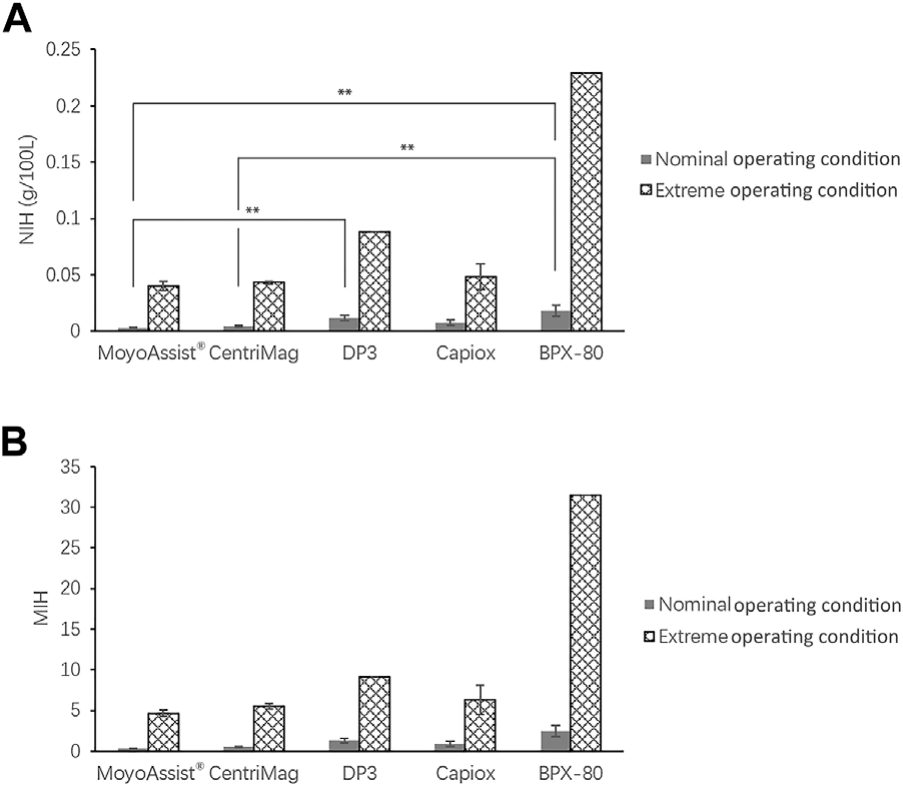
Averages of **(A)** NIH and **(B)** MIH for all the centrifugal blood pumps. The sample sizes were MoyoAssist^®^ (*n* = 3; *n* = 3), CentriMag (*n* = 3; *n* = 3), DP3 (*n* = 3; *n* = 1), Capiox (*n* = 3; *n* = 2), and BPX-80 (*n* = 2; *n* = 1), representing nominal operating conditions and extreme operating conditions. Porcine blood subjected to circulation loops for 6 h. Hemolysis was measured and calculated in accordance with ASTM. A value of *p* < 0.05 (**) was considered to be statistically significant.

The result of *in vitro* hemolysis in terms of NIH and MIH for all the test blood pumps is shown in Figure 2. The hemolysis values of all the test pumps at extreme operating conditions were 6–13 times higher than those of the nominal operating conditions. At both nominal and extreme operating conditions, the averages of NIH and MIH ranked from low to high were MoyoAssist^®^, CentriMag, Capiox, DP3, and BPX-80. There was a significant difference among the five devices at the nominal operating condition (*p* = 0.01), with a significant difference between MoyoAssist^®^ and DP3 (*p* = 0.03), MoyoAssist^®^ and BPX-80 (*p* = 0.02), and CentriMag and BPX-80 (*p* = 0.03). No significant difference was demonstrated among MoyoAssist^®^, CentriMag, and Capiox at extreme operating conditions. DP3 and BPX-80, on extreme operation, were not statistically comparable since they were only conducted for a single test. However, they presented approximately 1–4 folds higher levels of hemolysis than the other three devices.

### 3.2 Hematology analysis

**FIGURE 3:**
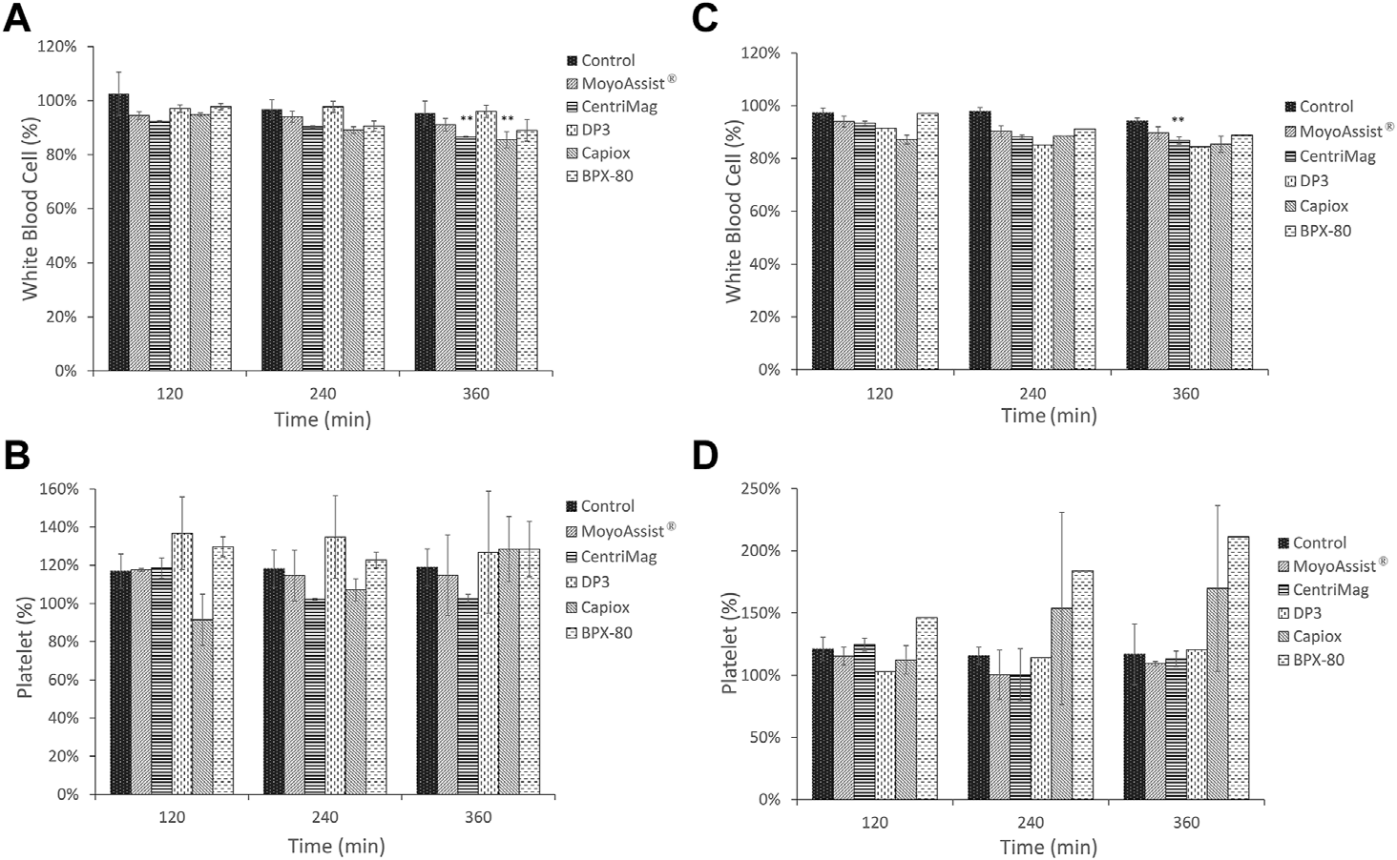
Comparison of temporal changes among control and centrifugal blood pumps on **(A)** white blood cells at nominal operating conditions, **(B)** platelets at nominal operating conditions, **(C)** white blood cells at extreme operating conditions, and **(D)** platelets at extreme operating conditions. The sample sizes were the same as those for the hemolysis test. Porcine blood was subjected to a circulation loop for 6 h and measured by automatic hematology analysis every 2 h. Results expressed as mean ± SD, % relative to each time zero samples. A value of *p* < 0.05 (**) was considered to be statistically significant.

The result of white blood cell and platelet counts every 2 hours for all the test blood pumps is shown in Figure 3. Due to the differences in the data baseline of blood samples, the ratios of each sample to the sample at time zero were presented. On nominal operation, there was a significant decrease in the white blood cell count over time for CentriMag and Capiox (*p* < 0.05) (Figure 3A). The number of white blood cells was reduced by more than 10% for both of them after 6-h circulation. No significant decrease in white blood cell numbers over time was observed for the other three devices. On extreme operation, there was a significant decrease over time for all the blood pumps (*p* < 0.05) (Figure 3C). The number of white blood cells was reduced by 10%–15% after a 6-h *in vitro* hemolysis test under extreme operating conditions. It indicated more damage to white blood cells at a low flow rate and high pressure head. In addition, no significant difference in white blood cell numbers was observed between the static control sample and blood pump samples at 360 min for MoyoAssist^®^ and Capiox. Since Capiox showed a significant decrease in white blood cell count over time on nominal operation, MoyoAssist^®^ performed best among the five devices at white blood cell counting. For the platelet count, there was no significant change over the testing period for all the blood pumps at both nominal and extreme operating conditions (Figures 3B, D). There was also no significant difference in platelet numbers between the static control sample and all the blood pump samples at 360 min.

### 3.3 The degradation of high-molecular-weight VWF

**FIGURE 4:**
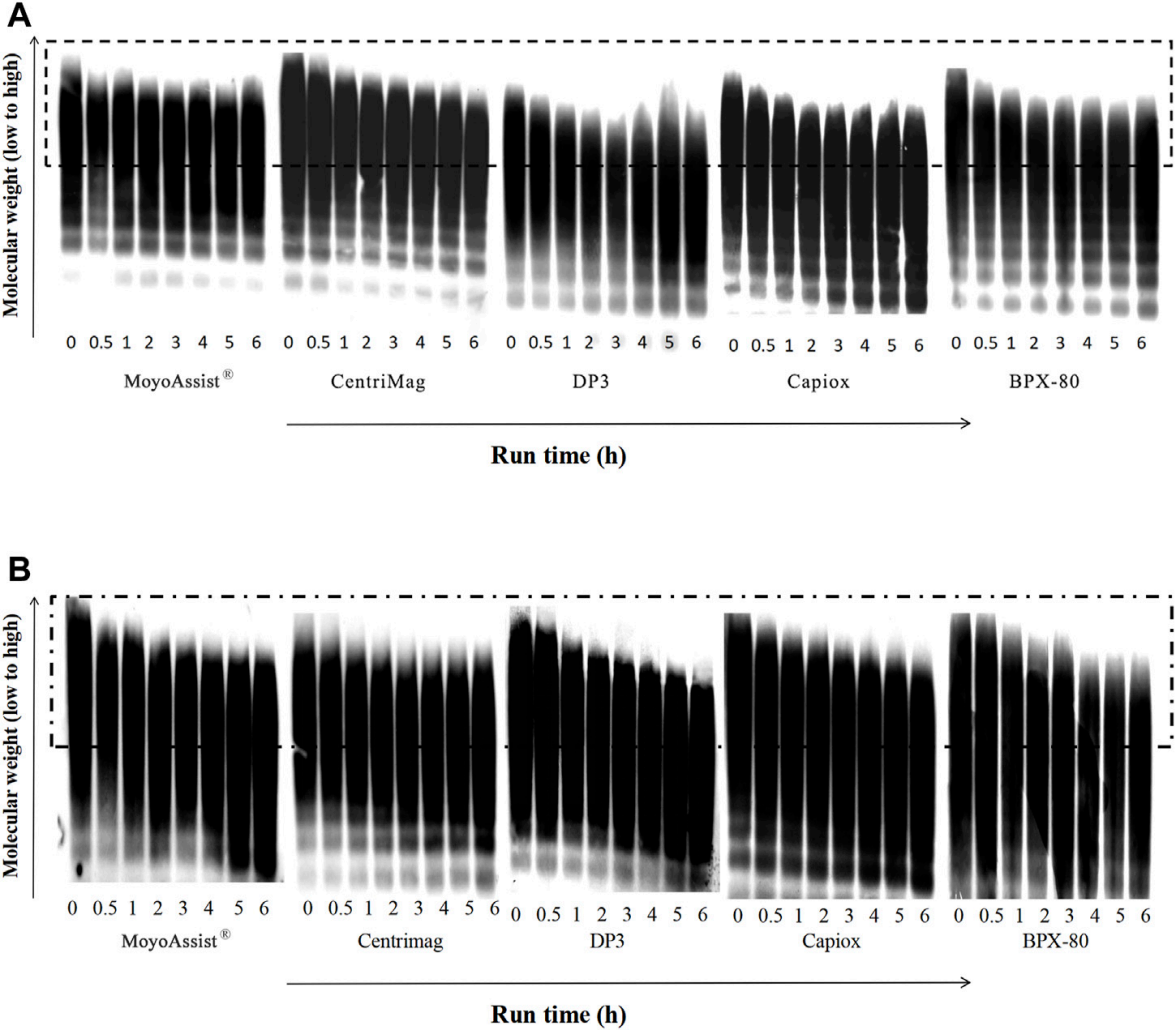
Representative immunoblot gel image of VWF multimers for all the centrifugal blood pumps at **(A)** nominal operating conditions and **(B)** extreme operating conditions. The sample sizes were the same as those for the hemolysis test. Porcine blood was subjected to circulation loops for 6 h and then measured by immunoblotting.

**FIGURE 5:**
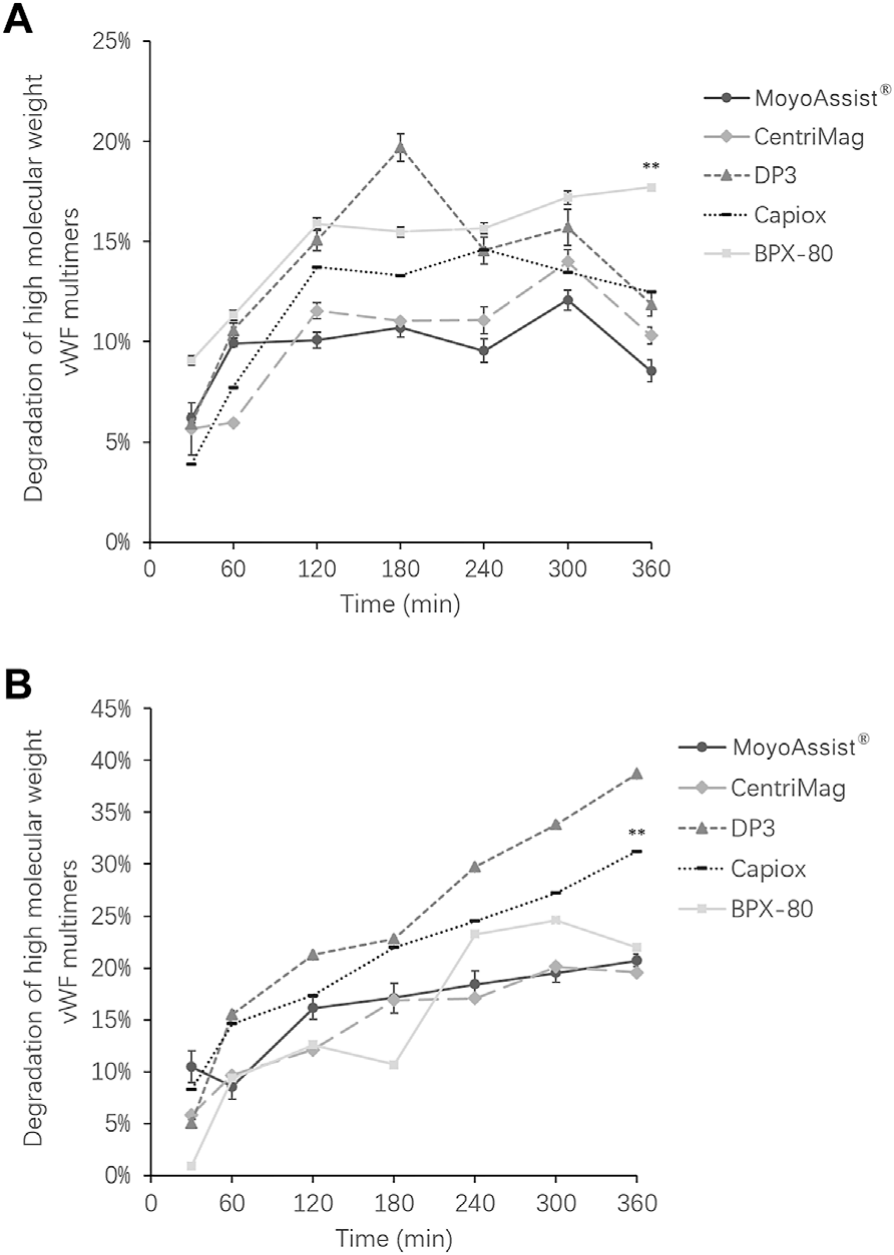
Degradation of high-molecular weight VWF multimers for all the centrifugal blood pumps at **(A)** nominal operating conditions and **(B)** extreme operating conditions. Results expressed as mean ± SD, % relative to each time zero sample. A value of *p* < 0.05 (**) was considered to be statistically significant.

For the study of high-molecular-weight VWF degradation, blood samples at time points 0, 30, 60, 120, 180, 240, 300, and 360 min were tested. During the 6-h test, a part of the high-molecular-weight VWF cut off to be small molecules under high shear stress. The representative image of VWF multimer bands obtained by immunoblotting is shown in Figure 4. The section in the dotted box represented the high-molecular-weight VWF multimer bands. The result indicated a gradual loss of high-molecular-weight VWF multimers over time for all the test blood pumps at both nominal and extreme operating conditions. Based on the representative images, the degradation of high-molecular-weight VWF multimers (%) was calculated (Eq. 4) and is shown in Figure 5. On nominal operation, the most severe degradation of VWF occurred in the first 2 h of the experiment (Figure 5A). It started to fluctuate after that. The degradation of high-molecular-weight VWF multimers was less than 20% during the entire test period. There was a significant difference among the five devices at the time point 360 min (*p* = 0.001). BPX-80 demonstrated a significant difference with the other four devices (*p* < 0.05), with at least 6% more degradation of high-molecular-weight VWF. On extreme operation, there was a significant increase in high-molecular-weight VWF degradation over time for all the test blood pumps (Figure 5B). Meanwhile, all the degradation of high-molecular-weight VWF multimers was more than 20% during the entire test period. It was obviously more severe than the VWF damage under nominal operation. There was a significant difference among MoyoAssist^®^, CentriMag, and Capiox at time point 360 min (*p* = 0.001). Capiox demonstrated a significant difference with the other two devices (*p* < 0.05), with 10% more degradation of high-molecular-weight VWF.

## 4 Discussion

In this study, the blood damage caused by four commercial extracorporeal centrifugal blood pumps widely used in clinics was comprehensively investigated. Experimental research on the influence of different operating conditions was conducted. The blood compatibility of a full maglev extracorporeal blood pump in development was also evaluated. All the centrifugal blood pumps were operated at nominal operating conditions (5 L/min, 160 mmHg) and extreme operating conditions (1 L/min, 290 mmHg) for clinical relevance. We did not consider conducting the experiments for right-sided support conditions (a high flow rate and low-pressure head) because the hemolysis will be lower under a lower pressure head. We considered the results under the worst case (a low flow rate and high-pressure head) more valuable. Hemolysis, calculated by the amount of plasma-free hemoglobin, is a reliable marker for red blood cell damage. The variable coefficient of NIH and MIH could be acceptable as in other certified labs. The hemolysis results of commercial pumps were consistent with the data in the literature (Hijikata et al., 2008; Schibilsky et al., 2020). MoyoAssist^®^ and CentriMag demonstrated lower hemolysis levels might be due to their non-contacting bearings associated with lower shear stress in pumps. The reason for the lower hemolysis of Capiox than DP3 and BPX-80 may be the lower operating pump speed (max. 3,000 rpm) that cause lower shear stress than the other two devices. White blood cells and platelets play an essential role in infection and thrombosis. Thus, their damage could contribute to adverse events in the clinic. The phenomenon of slightly increased platelet counts (false-positive) in the automatic hematology analyzer has been confirmed to be associated with the release of microparticles from white blood cells (Radley et al., 2019).

It is known that the VWF damage caused by centrifugal blood pumps is a major factor in GI bleeding. Under non-physiological shear stress, the high-molecular-weight multimers of VWF will easily be degraded into small molecules and thus cannot participate in the coagulation process. The high-molecular-weight VWF decreased by less than 20% during the test period for all the blood pumps on nominal operation, which is consistent with the published data on CentriMag. The result indicated that the loss of high-molecular-weight VWF over time exists in all the centrifugal blood pumps; thus, it needs to be paid attention to in preclinical testing. Interestingly, BPX-80, which performed much worse than the other blood pumps in hemolysis, demonstrated low VWF damage at extreme operation. This phenomenon may be related to the different damage mechanisms of red blood cells and VWF (Mei et al., 2022a; Mei et al., 2022b). The shear stress threshold for the damage to VWF was much lower than the damage to red blood cells (Nascimbene et al., 2016). Other factors also affect the degradation of high-molecular-weight VWF besides high shear stress, such as a surface that easily adheres to VWF and unfolds the chain segments. Therefore, the blood pump with higher shear stress may cause more hemolysis but not necessarily yield more damage to VWF. In addition, the rate of VWF degradation was the fastest at the beginning, followed by a gradual decline. It suggested that VWF damage induced by non-physiological shear stress is rapid and irreversible.

Comparing the *in vitro* hemocompatibility of blood pumps at different operations, the blood damage was more severe at extreme operating conditions than that at nominal operating conditions. It was consistent with the published data (Chan et al., 2015). At the same flow rate, the hemolysis index increased with the increase of pressure head. In addition, the hemolysis index decreased with the increase in flow rate at the same pressure head. Another research showed lower hemolysis in pediatric LVAD operating conditions than in adult LVAD operating conditions, with about half of the flow rate and 70% of the pressure head (Kuck et al., 2021). The order of most to least hemolysis remained the same as the nominal operating condition. However, the order of most to least VWF damage was not the same at different operations. This may also be related to the different damage mechanisms of red blood cells and VWF, as mentioned in the previous paragraph. The more severe blood damage in blood pumps at extreme operating conditions could be associated with more adverse events in clinics. Therefore, special attention should be paid to the hemocompatibility performance of extracorporeal centrifugal blood pumps at extreme operation to reduce adverse events in clinical practice.

Third-generation VAD utilizing non-contacting bearings with hydrodynamic suspension and/or magnetic levitation technology has been developed to reduce blood damage-associated adverse events. Compared to mechanical bearing, a non-contacting bearing allows for a bigger secondary flow path and, thus, lower shear stress distributed in the blood pump. Therefore, pumps with non-contacting bearings were supposed to perform better than those with mechanical bearings in *in vitro* hemocompatibility. CentriMag and MoyoAssist^®^ involved in this study are magnetically levitated centrifugal pumps, while Capiox, DP3, and BPX-80 are mechanical bearing centrifugal pumps. The results demonstrated the superior hemocompatibility of CentriMag and MoyoAssist^®^, with overall low blood damage at the hemolysis level, blood cell counts, and degradation of high-molecular-weight VWF. They were expected to reduce adverse events associated with blood damage in clinical practice.

The CentriMag centrifugal blood pump has a long successful history of clinical use and caused low blood damage during *in vitro* testing (Zhang et al., 2006; Chan et al., 2015). In this study, as the only pump in development, MoyoAssist^®^ demonstrated even lower white blood cell damage than CentriMag. The findings suggested that non-contacting bearings with magnetic levitation have an absolute advantage in comprehensive hemocompatibility compared to mechanical bearings in blood pumps. Since the MoyoAssist^®^ blood pump shows good performance in *in vitro* blood damage testing, it has great potential in the future.

In addition, the blood circulation loop built in this study consisted of 1 m of 3/8″-diameter tubing. The total blood volume in the loop was 310 ± 10 mL. It could be considered as a downscaled test loop with a lower priming volume according to the old version of the ASTM standard (2017). However, a mini test-loop was proved to significantly better differentiate the blood pumps with fewer adverse effects by the loop itself (Woelke et al., 2020). A 160-mL test loop provided significantly higher plasma-free hemoglobin increase and consistently stronger VWF degradation than a 480-mL test loop. Therefore, to test as many circuits as possible simultaneously, a downscaled circulation loop may be useful to evaluate *in vitro* blood damage.

## 5 Limitations and future directions

In this study, all the centrifugal blood pumps were evaluated by *in vitro* models with well-controlled experimental conditions. Clinical scenarios integrate a plethora of variables, especially patient-related, which are associated with phenomena such as hemolysis, leukopenia, and altered hemostasis. Therefore, the results from an *in vitro* study may be different from clinical practice. In addition, the low sample size was due to the limited pump heads and the small number of animals as the blood source. Repeated tests at extreme operating conditions were not conducted for DP3 and BPX-80. In addition, all the centrifugal blood pumps were tested at operating conditions for the extracorporeal VAD application. In the future, the *in vitro* blood damage tests will be conducted at ECMO operating conditions to reflect the hemocompatibility for cardiopulmonary support application. The platelet activation will also be measured to evaluate the risk of clot formation.

## 6 Conclusion

In this study, the hemocompatibility of five extracorporeal centrifugal blood pumps was investigated comprehensively. Compared to mechanical bearing blood pumps, the magnetically levitated centrifugal pumps CentriMag and MoyoAssist^®^ demonstrated an advantage in hemocompatibility under both nominal and extreme operations. As a pump in development, the MoyoAssist^®^ blood pump has good *in vitro* hemocompatibility. In addition, the blood damage was more severe at extreme operating conditions than that at nominal operating conditions. The performance of the five blood pumps was arranged in different orders at these two operating conditions. Therefore, including multiple operating conditions of blood pumps in *in vitro* hemocompatibility evaluation will be helpful for clinical application.

## Data Availability

The original contributions presented in the study are included in the article/supplementary material; further inquiries can be directed to the corresponding authors.
